# Using the alternative model of personality disorders for DSM-5 traits to identify personality types, and the relationship with disordered eating, depression, anxiety and stress

**DOI:** 10.1186/s40337-025-01204-2

**Published:** 2025-02-07

**Authors:** Tanya Gilmartin, Caroline Gurvich, Joanna F. Dipnall, Gemma Sharp

**Affiliations:** 1 Department of Neuroscience, Monash University and the Alfred Hospital, Melbourne, Australia; 2 Monash Alfred Psychiatry Research Centre, Monash University and The Alfred Hospital, Melbourne, Australia; 3 School of Public Health and Preventive Medicine, Monash University, Melbourne, 3004 Australia; 4 Institute for Mental and Physical Health and Clinical Translation, School of Medicine, Deakin University, Geelong, 3220 Australia; 5 School of Psychology, University of Queensland, 4067 St Lucia, QLD Australia

**Keywords:** Eating disorders, Disordered eating, Personality types, Overcontrol, Undercontrol, PID-5

## Abstract

**Background:**

There is a substantial and growing evidence base that has identified three distinct personality types (Overcontrol, Undercontrol and Resilient) among samples of individuals with eating disorders, as well as non-clinical samples. Even in studies where up to six personality types have been identified, the three core types representing Overcontrol, Undercontrol and Resilient consistently emerge. The aim of the research was to explore whether latent Overcontrol and Undercontrol personality types could be identified using pathological personality types as part of the Alternative Model for Personality Disorders published in DSM-5. We further aimed to understand how these personality types were associated with eating pathology, depressed mood and anxiety.

**Methods:**

A total of 391 women, 167 men and 10 gender-diverse individuals aged 16 to 31 years completed measures of the alternative model of personality disorder traits, disordered eating behaviours, eating pathology, depression, anxiety and stress. A systematic four-step process using hierarchical, k-means, and random forest cluster analyses were used to identify the best fitting cluster solution in the data.

**Results:**

The results revealed a four-cluster solution that represented overcontrol, undercontrol, resilient and an antisocial/psychoticism cluster. The overcontrol, undercontrol, and antisocial/psychoticism types were all associated with increased disordered eating, eating pathology, depression, anxiety and stress compared to the resilient types, with the undercontrol cluster scoring significantly higher than the other three clusters on all measures of clinical pathology.

**Conclusions:**

Pathological personality traits, as conceptualised within the DSM-5 alternative model of personality disorders may have merit for identifying overcontrol and undercontrol personality types. Our findings provide additional evidence that both overcontrol and undercontrol personality types are associated with increased eating pathology, depression, anxiety and stress.

## Abstract (plain language summary)

Understanding eating disorders in the context of overcontrol, undercontrol and resilient personality types has been found to predict disordered eating behaviour and treatment course. More contemporary models of personality disorder tend to consider personality and personality disorders as a series of continuous traits. Our research aimed to understand how twenty-five pathological personality traits might define personality types amongst a group of young people in Australia. Three broad personality types have been identified consistently in previous research, being overcontrol (rigid, perfectionistic), undercontrol (impulsive, mood dependent) and resilient (flexible, low pathology). Our study identified these types within our data along with a fourth, antisocial/psychoticism (attention seeking, risk taking) cluster that had not previously been identified. It was further found that individuals who were in the overcontrol, undercontrol or antisocial/psychoticism groups were more likely to experience eating pathology and disordered eating, as well as depression, anxiety and stress compared to resilient types. Overall, this means that that the alternative, dimensional model of personality pathology may be useful for identifying people at risk for developing an eating disorder and to consider treatment needs.

## Background

Initially documented in developmental literature [1–4], there has been an increasing research and clinical focus on the presence of personality types in research and clinical practice [5, 6, 7]. The contemporary understanding of personality types is that Overcontrol (OC) and Undercontrol (UC) represent multidimensional constructs that form a continuum, with maladaptive variants being affiliated with each pole, and a resilient group that exists in the middle. OC is characterised by rigidity, perfectionism and interpersonal avoidance, while UC is associated with impulsivity and dysregulation [7–11]. The resilient type represents adaptive or flexible variants of OC and UC and is associated with less personality and clinical dysfunction compared with the OC and UC types [7].

There appears to be some clinical utility to the classification of individuals presenting with an ED based on personality types. OC and UC personality types have been associated with a 15.9 and 6.5 times higher risk of developing an ED, respectively [12]. Furthermore, OC and UC groups have been found to score higher on self-report measures of eating pathology and psychological distress compared to resilient types, with small and medium effect sizes, respectively [9, 13]. In addition, classifying individuals who have an ED as OC, UC or resilient has been found to be more predictive of longitudinal outcomes in ED treatment [6, 14].

There is considerable variation in clustering strategy and psychometric measures used by researchers, and as a result, although three distinct personality types tend to emerge from the data, additional groups have been found in some studies. For example, Thompson-Brenner and their team identified a five-cluster solution that comprised of a “avoidant-insecure” and the “obsessional-sensitive” groups that shared features of the OC group in other studies [14]. Their identified “behaviourally dysregulated” and “emotionally dysregulated” groups both shared features with UC clusters identified in other studies. A “high functioning” or resilient group was also identified [6, 10, 11, 14, 15]. In another study, a six-cluster solution was found to be the produced the best fit for the data. The “inhibited” and “impulsive” profiles resembled OC and UC respectively. Two profiles (labelled “self-focused” and “average”) resembled the resilient or low psychopathology profile of other studies [16]. The presence of two low-psychopathology groups may be explained by theoretical frameworks, specifically the “self-focused” group may represent an adaptive variant of UC, and the “average” group may represent an adaptive variant of OC [7]. Theoretically, the “resilient” cluster identified in other studies may represent a mixture of these groups.

Although there is a substantial evidence base indicating the relationship between personality types and EDs or disordered eating [6, 9, 12–14], there is a notable gap in the research literature that has tended to focus on assessing normative personality traits types in clinical samples [12, 17]. Whilst existing literature has laid important framework for understanding the personality types construct and relationship with EDs, measures of normative personality traits are rarely used in clinical practice, which may be a barrier to translating research into practice. A further concern is that focusing on identifying personality types in clinical samples may provide a biased or skewed representation of personality types [9].

The Diagnostic and Statistical Manual of Mental Disorders (DSM-5; 1) published an Alternative Model of Personality Disorder (AMPD) that was introduced as a dimensional alternative to the traditional categorical model as area for further research. The introduction of the AMPD represents an advancement of the understanding of personality and personality pathology as a dimensional construct [18–20]. The dimensional approach to personality pathology has provided increased opportunity to understand multi-dimensional constructs. For example, existing categorical personality disorders have been re-conceptualised as multi-dimensional constructs in the AMPD [21–29] and composites exist for identifying categorical PDs using AMPD pathological personality traits [30]. There is further opportunity to explore the relationship between dimensional measures of personality pathology that are designed for use in clinical practice, and constructs such as personality types.

Radically-Open DBT (RO-DBT) is a transdiagnostic treatment that has been developed to treat individuals who present with a maladaptive overcontrol personality type [7]. Of particular significance is the focus on treating underlying dysfunction resulting from the interaction between maladaptive OC behaviours and other people in the client’s lives, rather than the presenting clinical disorder (i.e. Depression, Anorexia Nervosa; 17). As part of the protocol, Lynch [7]. has redefined OC and UC constructs in clinical settings. He theorised that some personality disorders present as OC disorders, and other are characteristic of UC disorders. Based on Lynch’s theory [7], it would be assumed that AMPD traits that were associated with OC personality disorders (Obsessive-compulsive, Paranoid, Avoidant and schizoid) such as rigid perfectionism, perseveration, intimacy avoidance, restricted affectivity, anxiousness, withdrawal and anhedonia would be associated with maladaptive OC [21]. Additionally, given that the OC construct has been associated with chronic depression [31], it can be hypothesised that depressivity may be another dimension associated with OC. In contrast, AMPD traits that were associated with UC personality disorders (Borderline, Antisocial, Narcissistic and Histrionic) such as manipulativeness, callousness, deceitfulness, hostility, risk taking, impulsivity, irresponsibility, emotional lability, anxiousness, separation insecurity, depressivity, attention seeking and grandiosity [21] would be expected to be indicative of UC. It would be further expected that the resilient or adaptive OC and UC presentations would demonstrate lower scores on PID-5 dimensions. However, this is no empirical research exploring these associations. While there is currently no widely adopted measure of assessing personality types [8], understanding how broad measures of pathological personality traits relate to OC and UC constructs may serve to bridge two gaps in the current knowledge. First, understanding the potential feasibility of using a measure of the AMPD to assess personality types may act as a step towards the broader assessment of personality types in clinical practice. Second, empirical evidence to support Lynch’s theory [7] regarding OC and UC personality disorders and the traits associated with these disorders (described above).

The overall aim of this research was to improve the understanding of conceptualising eating and clinical pathology in the context of personality types. Although level of personality dysfunction is measured as part of the AMPD Criterion A [32], we decided to assess level of clinical dysfunction, more specifically eating pathology, mood, anxiety and stress.

Therefore, the present study has two specific aims. The first aim was to explore whether underlying clusters exist in a mixed gender, community sample that represent OC, UC and resilient personality types. It was expected that three or more clusters of participants would emerge in the data, reflecting adaptive and maladaptive OC and UC personality types, in line with past research [6, 10, 11, 14–16].

Second, we aimed to explore the relationship between cluster membership and clinical presentation. It was expected that maladaptive OC and UC groups would report increased clinical dysfunction as assessed using measures of eating pathology, depression, anxiety and stress.

## Methods

### Participants

Prior to study commencement, all procedures were approved by Monash University institutional ethics committee (Project ID: 25083). A total of 568 individuals completed all measures of the study. Prospective participants (*N* = 1,492) accessed information about the study from an online advertisement. A flowchart of participation and study drop-out has previously been published [9, 33, 34]. The age range of participants was 16–31 (M = 22.17, SD = 3.83), including 167 men (29.4%, M = 21.76, SD = 3.62), 391 women (68.8%, M = 22.35, SD = 3.93) and 10 individuals identified as gender diverse (1.8%, M = 21.8, SD = 3.05). The majority of participants were Caucasian (69%) and/or had a Year 12 (final year of high school) or equivalent education (45%). Further information about this sample has been previously published [33]. As sufficient statistical power can be achieved in relatively small samples [35] no approximate sample size was estimated prior to data collection for the cluster analysis.

### Measures

Participants completed a survey designed to measure DSM-5 pathological personality dimensions, eating behaviour and clinical symptoms between December 2020 and May 2021. All measures used in the current study have been validated in comparable samples [36–41].

### Personality

The measure of personality pathology that was selected for use in the current study was the PID-5 Short-Form (PID-5-SF; 50). This is a 100-item measure has been found to be highly correlated with the original scale [42]. The scale assesses the 25 AMPD lower-order pathological personality traits. The scale asks participants if items reflect how they may describe themselves (e.g. “I am easily angered,” “I’m always worrying about something”) and are scored on four-point Likert scale ranging from 0 (very false or often false) to 3 (very true or often true). Higher scores indicate higher pathology. The four items measuring each facet are summed and the mean computed for facet scores. In the current sample, majority of the subscales demonstrated adequate to strong internal consistency (α = 0.71–0.91) with the exception of Irresponsibility which demonstrated questionable internal consistency (α = 0.61). It was decided to retain the scale due to the exploratory nature of the study.

### Eating behaviour

The Eating Disorder Examination Questionnaire-Short (EDE-QS) asks participants to select on how many days they engaged in particular behaviours (e.g., “Have you had a strong desire to lose weight?” “Have you had a sense of having lost control over your eating”) and represents a 12-item version of the original Eating Disorder Examination Questionnaire (EDE-Q; 51). The items are with items are scored on a 4-point Likert scale ranging from 0 (0 days) to 3 (6–7 days). The scores obtained on each item are added together to achieve a total score ranging from 0 to 36 that represents eating pathology [43]. Scores of 15 and above have been found to be indicative of the presence of an eating disorder [44]. A Cronbach’s alpha coefficient of α = 0.91 was found in the current sample, indicating strong internal consistency. The original version of the EDE-Q has been found to be appropriate for use with males and females [45].

The Eating Pathology Symptoms Inventory (EPSI; 54) was used to assess disordered eating behaviour. This is a 45-item scale that consists of eight subscales, with Body Dissatisfaction (e.g., “I did not like how clothes fit the shape of my body”) providing a measure of core eating and weight concerns, and seven subscales designed to measure specific disordered eating behaviours (Restriction, Binge eating, Purging, Cognitive Restraint, Negative Attitudes Towards Obesity, Excessive Exercise and Muscle Building). An additional item was included as part of the scale to measure chewing and spitting behaviour (“I spat out food after chewing to avoid putting on weight”) based on surveys administered by [46] and worded to remain consistent with the other items in the EPSI. The EPSI is scored on a five-point Likert scale ranging from 0 (Never) to 4 (Very often), and the items for each subscale are summed together to obtain a total score, with the score range for each subscale varying based on different number of items (e.g. Body dissatisfaction score range 0–28, Cognitive restraint score range 0–12). The internal consistency in the current study was found to range from good to strong (α = 0.79–0.90). The EPSI has previously been found to have good test-retest reliability for all scales for men and women together, in addition to being invariant across gender [47], and for most scales when genders were considered separately [48].

### Depression, anxiety and stress

Depression, anxiety and stress were measured by the Depression Anxiety Stress Scale-21 item version (DASS-21; 57). The Depression subscale measures low positive affect (e.g., “I couldn’t seem to experience any positive feeling at all”), Anxiety measures physical hyperarousal (e.g., “I felt I was close to panic”) and Stress measures tension or irritability (e.g. “I found it difficult to relax”; 58). The DASS-21 is scored on a 4-point Likert scale ranging from 0 (Did not apply to me at all/Never) to 4 (Applied to me very much or most of the time/Almost Always), with the item scores for each subscale summed to obtain total scores ranging from 0 to 28, and then multiplied by two to obtain scores consistent with the original 42-item version (Scores ranging from 0 to 56; 57). Research has replicated the three-factor structure of the DASS and DASS-21 [49, 50] and the DASS has demonstrated good internal consistency in our sample (α = 0.84–0.91).

### Procedures

Data from this study has previously been described elsewhere [9, 33, 34]. Prospective participants accessed study information regarding the goals and anticipated time commitment of the study on social media and made informed consent about the participation in the study. The posts were targeted towards young adults and were posted on social media pages and electronic newsletters associated with eating disorder and personality disorder organisations, university pages, community and sport notice boards, and pages associated with interest groups such as fitness, trades and food. The questions in the survey were designed to collect demographic information in addition to the above-described psychometric measures. At the completion of the survey, participants were provided with the opportunity to enter a draw to win a voucher worth $50AUD.

### Statistical analysis

**Fig. 1:**
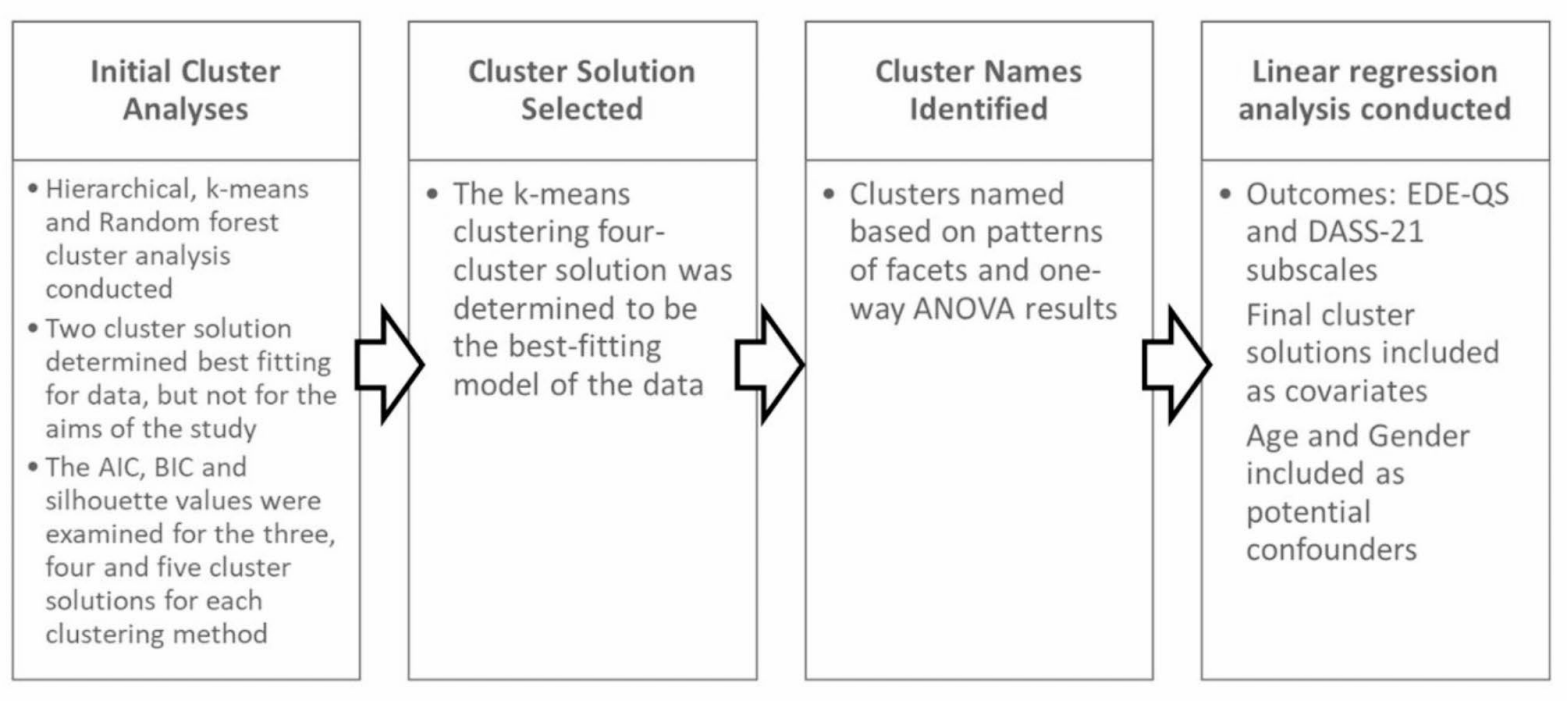
Data analysis flowchart

The JASP statistical package version 0.14.1 [51] was used to conduct the cluster analyses. SPSS version 27 [52] was the statistical package used for all other calculations. The statistical analysis process has been outlined as Fig. 1. All 25 PID-5-SF facets were entered into a Hierarchical, k-means and random forest cluster analyses. The Ward’s method was used for the hierarchical cluster analysis with squared Euclidean distance to systematically group similar respondents together to form clusters [53]. For the k-means cluster analysis, each cluster consists of similar participants using the Hartigan-Wong algorithm, with a maximum of 25 iterations for a defined number of clusters [54]. The random forest algorithm develops decision trees to group individual cases together with the algorithm set at 1000 decision trees [55]. Initially, the a two-cluster solution was found to be optimal for the Hierarchical and k-means cluster analyses using the silhouette value [56]. Cluster 1 demonstrated high scores on all facets, and was found to represent a high psychopathology group, and cluster 2 represented a low psychopathology group. However, since the aim of the current study was to explore potential facet-level differences, and to understand the nuances of maladaptive personality presentations, three, four and five cluster solutions were also examined. For cluster solutions 3 to five, the silhouette value in addition to the Akaike Information Criterion (AIC; 65) and Bayesian Information Criterion (BIC; 66) were used to determine the best fitting solution for the data. The AIC and BIC are commonly used as model selection criterions, where the lowest value represents the better model [57, 58]. The k-means clustering four-cluster solution was determined to be the best-fitting model of the data (see Table 1).

The naming of the four-cluster solution was informed by the pattern of means of the input variables. The differences between clusters on PID-5-SF pathological domains, EDE-QS, EPSI and DASS-21 mean scores were examined using one-way Analysis of Variance (ANOVAs), the frequency of clinical significance of EDE-QS scores were compared between clusters using a chi-squared tests of association. A multivariable linear regression was conducted to assess the extent to which cluster membership predicted score on the EDE-QS or the DASS-21 subscales. Age and gender were included in the analysis because eating disorders are more likely to emerge in adolescence than adulthood with individuals younger in age have been found to be more likely to restrict eating [21, 33]. In addition, females have been found to experience eating disorders at a higher frequency than males [21, 59].

## Results

Table 1 displays the silhouette value, AIC and BIC values for each of the cluster solutions. The k-means four and five-cluster solutions were examined as demonstrating the most desirable statistical metrics. Whilst the five-cluster solution demonstrated lower AIC and BIC values, the solution also consisted of two clusters with < 100 participants. The four-cluster solution was considered a more meaningful parsimonious solution in the broader context of our study. Therefore, the k-means four-cluster also solution provided an option that balanced lower AIC and BIC values compared to two and three cluster solutions, and higher silhouette value compared to five-cluster solutions.”

**Table 1:** The AIC, BIC and silhouette scores for the hierarchical, k-means and Random Forest Cluster strategies, for two, three, four and five cluster solutions

Cluster strategy	AIC	BIC	Silhouette
**Two-cluster solution**			
Hierarchical	11915.77	12132.88	0.13
k-means	**11219.70**	**11436.80**	**0.19**
Random forest	12866.59	13083.700	0.08
**Three-cluster solution**			
Hierarchical	10810.47	11136.13	0.09
k-means	**10402.27**	**10727.93**	**0.11**
Random forest	12297.38	12688.17	**0.11**
**Four-cluster solution**			
Hierarchical	10382.40	10816.62	0.09
k-means	**9829.74**	**10263.95**	**0.12**
Random forest	10957.44	11391.65	0.01
**Five-cluster solution**			
Hierarchical	10031.09	10573.86	0.08
k-means	**9426.91**	**9969.67**	**0.11**
Random forest	10287.70	10830.47	0.04

Note. Optimal solution for each of the two, three, four and five-cluster solutions is indicated in **bold.** AIC = Akaike Information Criterion BIC = Bayesian Information Criterion

The demographic details for each cluster have been displayed in Table 2, and the means and standard deviations for each of the PID-5 subscales, in addition to the EPSI, DASS-21 and EDE-QS, in addition to one-way ANOVAs to assess the differences between clusters have been displayed in Table 3.

**Table 2:** Age and gender for each cluster

Cluster	*N*	Age (M, SD)	Men (*N*, %)	Women (*N*, %)	Non-Binary (*N*, %)
Cluster 1 (Maladaptive OC)	155	22.42, 3.89	26, 16.8%	126, 81.3%	3, 1.9%
Cluster 2 (Maladaptive UC)	139	21.84, 3.789	32, 23%	104, 74.8%	3, 2.2%
Cluster 4 (Resilient)	156	22.67, 3.93	53, 34.0%	101, 64.7%	2, 1.3%
Cluster 4 (Antisocial/Psychoticism)	118	21.54, 3.59	56, 47.5%	60, 50.8%	2, 1.7%

**Table 3:**
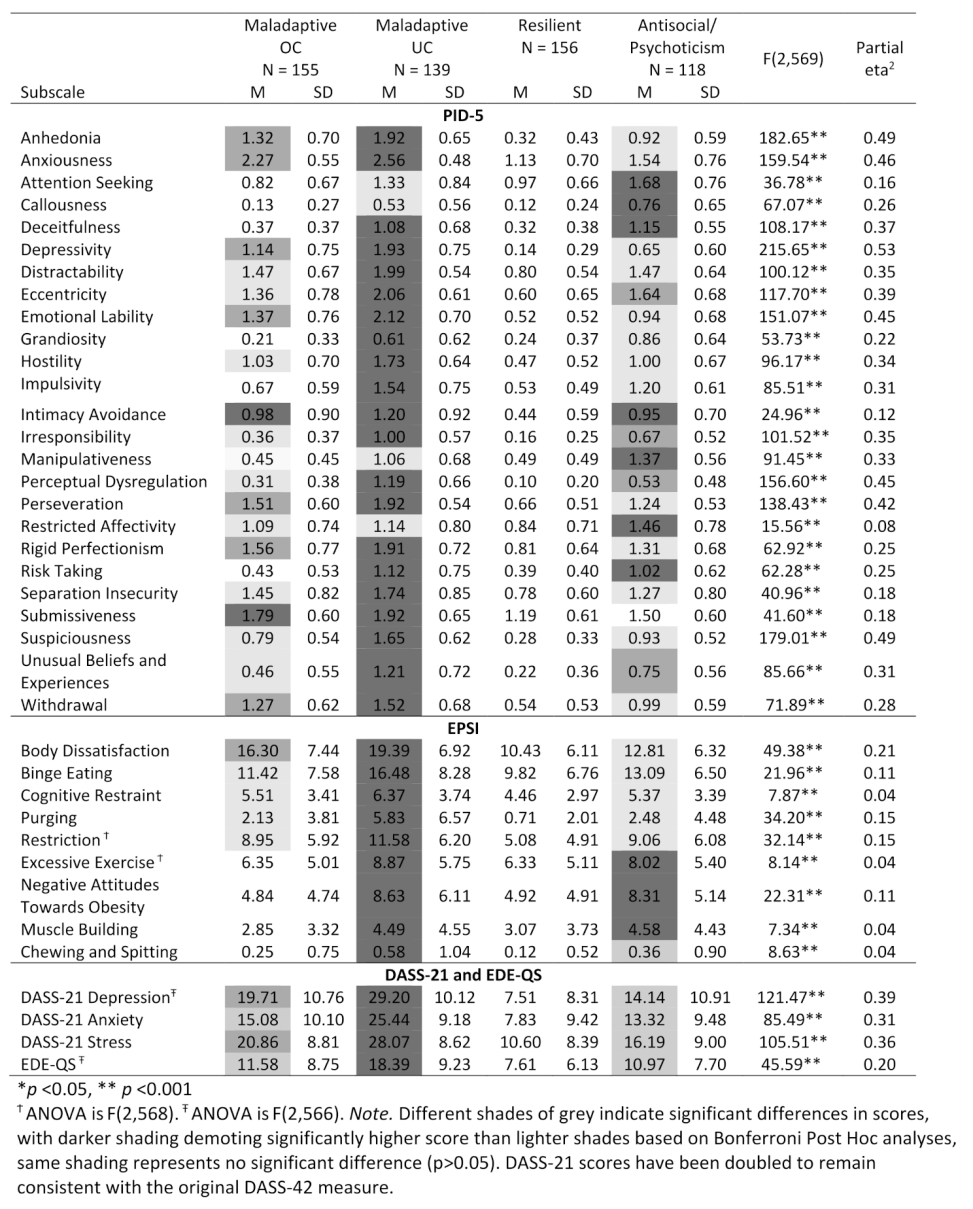
Means, standard deviations and one-way ANOVA results for the PID-5, EPSI, DAS-21 subscales and EDE-QS

Cluster 1 (27.29% of the sample) was identified as the maladaptive OC cluster and was characterised by higher scores than other clusters on intimacy avoidance and submissiveness in addition to elevated scores on the anhedonia, anxiousness, depressivity, emotional lability, perseveration, rigid perfectionism, and withdrawal. This cluster was further characterised by low scores on attention seeking, callousness, deceitfulness, impulsivity, manipulativeness and risk taking compared to the maladaptive UC and antisocial/psychoticism cluster. Further, the maladaptive OC cluster/Cluster 1 demonstrated elevated mean scores of EPSI body dissatisfaction and DASS-21 depression and stress. According to normative data, DASS-21 depression and Stress were in the moderate range whilst anxiety was severe [60].

Cluster 2 (24.47% of the sample) demonstrated scores higher than other clusters on most PID-5 subscales, except attention seeking, callousness, manipulativeness and restricted affectivity in addition to high scores on the EPSI, DASS-21 and EDE-QS and is labelled the high maladaptive UC cluster. The mean scores for DASS-21 depression and anxiety were in the extremely severe range, while stress was in the severe range compared to norms [60]. Cluster 3 (27.46% of the sample) was characterised by low scores on all measured subscales compared to the other three clusters and was named the resilient cluster. Individuals in the resilient cluster were found to achieve mean scores in the normal range for measures of DASS-21 depression, anxiety and stress compared to norms [60].

Cluster 4 (20.77% of the sample) was characterised by scores on the attention seeking, callousness, deceitfulness, intimacy avoidance, manipulativeness, restricted affectivity and risk taking subscales that was higher than the other clusters. In addition, elevated scores were found on the eccentricity, irresponsibility, perceptual dysregulation and unusual beliefs and experiences scales, and low scores were found on the submissiveness subscale. This cluster was named the antisocial/psychoticism cluster. Regarding the clinical sales, low-moderate scores were obtained on all the EPSI, DASS-21 and EDE-QS scales, with the exception of Excessive Exercise, Negative Attitudes Towards Obesity, and Muscle Building where a significantly higher score was achieved compared to the maladaptive OC or resilient cluster. The average scores for the antisocial/psychoticism clusters for DASS-21 depression and anxiety were in the moderate range, and stress was considered mild [60].

### Predicting eating pathology, depression, anxiety and stress

The frequencies of participants who achieved a clinical EDE-QS score by cluster have been displayed in Table 4. A chi-square test of independence was performed to examine the relationship between cluster solution and clinical EDE-QS score. Three participants completed the PID-5-SF but not the EDE-QS and were therefore included in the cluster analyses but excluded from the chi-squared analysis. There was a significant association between cluster group and EDE-QS (Χ^2^( *N* = 565) = 87.84, *p* < 0.001). Individuals in the high psychopathology cluster were found to be more likely to achieve clinical scores on the EDE-QS than expected, whilst those in the resilient cluster were less likely to achieve clinical scores than expected.

**Table 4:** Frequency and percentage of clinical EDE-QS score by personality type

Cluster	*N*	EDE-QS score < 15 *N* (%)	EDE-QS score >/=15 *N* (%)
Maladaptive Overcontrol (OC)	155	97 (63.4%)	56 (36.6%)
Maladaptive Undercontrol (UC)	139	45 (32.4%)	94 (67.6%)
Resilient	156	132 (85.2%)	23 (14.8%)
Antisocial/Psychoticism	118	77 (61.2%)	41 (34.7%)

*N* = 565

Results of the multivariable linear regression analyses to explore cluster solutions being a predictor of eating pathology, depression, anxiety and stress are presented in Table 5.

**Table 5:** Results of Linear regression analysis using cluster membership to Predict Eating Pathology, Depression, anxiety and stress

	Predictor	b	SE	beta	t	*p*-value	Zero-Order	Part *r*	Partial *r*
	**EDE-QS**								
Block 1	(Constant)	18.39	0.68		27.01	**< 0.001**			
	OC^1^	-6.81	0.94	-0.34	-7.25	**< 0.001**	-0.03	-0.27	-0.29
	UC^1^	-10.78	0.94	-0.54	-11.49	**< 0.001**	-0.31	-0.44	-0.44
	Antisocial/Psychoticism^1^	-7.42	1.01	-0.34	-7.39	**< 0.001**	-0.06	-0.28	-0.30
Fit	R^2^ = 0.19, F(3,561) = 45.59, *p* < 0.001								
Block 2	(Constant)	16.50	2.08		7.95	**< 0.001**			
	OC^1^	-7.01	0.93	-0.35	-7.56	**< 0.001**	-0.03	-0.28	-0.31
	UC^1^	-10.42	0.93	-0.52	-11.21	**< 0.001**	-0.31	-0.42	-0.43
	Antisocial/Psychoticism^1^	-6.63	1.01	-0.30	-6.60	**< 0.001**	-0.06	-0.25	-0.27
	Age	-0.03	0.09	-0.01	-0.32	0.753	0.21	-0.01	-0.01
	Female^2^	3.37	0.76	0.18	4.46	**< 0.001**	-0.05	0.17	0.19
	Gender Diverse^2^	-1.39	2.58	-0.02	-0.54	0.590	-0.03	-0.02	-0.02
Fit	R^2^ = 0.22, F(6,558) = 27.26, *p* < 0.001								
Difference	Δ R^2^ = 0.03, *p* < 0.001								
	**DASS-21 Depression**								
Block 1	(Constant)	29.20	0.85		34.26	**< 0.001**			
	OC^1^	-9.49	1.17	-0.33	-8.08	**< 0.001**	0.11	-0.27	-0.32
	UC^1^	-21.69	1.17	-0.76	-18.54	**< 0.001**	-0.48	-0.61	-0.62
	Antisocial/Psychoticism^1^	-15.07	1.26	-0.48	-11.97	**< 0.001**	-0.13	-0.39	-0.45
Fit	R^2^ = 0.39, F(3,561) = 121.47, *p* < 0.001								
Block 2	(Constant)	28.83	2.63		10.95	**< 0.001**			
	OC^1^	-9.44	1.18	-0.33	-8.02	**< 0.001**	0.11	-0.26	-0.26
	UC^1^	-21.93	1.18	-0.77	-18.60	**< 0.001**	-0.48	-0.61	-0.61
	Antisocial/Psychoticism^1^	-15.42	1.28	-0.49	-12.06	**< 0.001**	-0.13	-0.40	-0.40
	Age	0.07	0.11	0.02	0.65	0.519	0.04	0.02	0.03
	Female^2^	-1.52	0.96	-0.06	-1.58	0.114	0.00	-0.05	-0.07
	Gender Diverse^2^	-2.89	3.27	-0.03	-0.89	0.376	-0.02	-0.03	-0.04
Fit	R^2^ = 0.39, F(6,558) = 61.28, *p* < 0.001.								
Difference	Δ R^2^ = 0.00, *p* = 0.371								
	**Dass-21 Anxiety**								
Block 1	(Constant)	25.44	0.81		31.36	**< 0.001**			
	OC^1^	-10.36	1.12	-0.40	-9.27	**< 0.001**	-0.01	-0.32	-0.36
	UC^1^	-17.61	1.12	-0.68	-15.78	**< 0.001**	-0.40	-0.55	-0.55
	Antisocial/Psychoticism^1^	-12.12	1.20	-0.43	-10.12	**< 0.001**	-0.09	-0.35	-0.39
Fit	R^2^ = 0.31, F(3,564) = 85.49, *p* < 0.001								
Block 2	(Constant)	28.25	2.51		11.25	**< 0.001**			
	OC^1^	-10.29	1.12	-0.40	-9.18	**< 0.001**	-0.01	-0.32	-0.36
	UC^1^	-17.43	1.13	-0.68	-15.50	**< 0.001**	-0.40	-0.54	-0.55
	Antisocial/Psychoticism^1^	-12.06	1.22	-0.43	-9.90	**< 0.001**	-0.09	-0.35	-0.39
	Age	-0.14	0.11	-0.05	-1.36	0.176	0.06	-0.05	-0.06
	Female^2^	0.37	0.92	0.02	0.40	0.688	0.03	0.01	0.02
	Gender Diverse^2^	2.06	3.12	0.02	0.66	0.510	-0.08	0.02	0.03
Fit	R^2^ = 0.31, F(6,561) = 43.07, *p* < 0.001.								
Difference	Δ R^2^ = 0.00, *p* = 0.512								
	**DASS-21 Stress**								
Block 1	(Constant)	28.07	0.74		38.09	**< 0.001**			
	OC^1^	-7.21	1.02	-0.30	-7.10	**< 0.001**	0.12	-0.24	-0.29
	UC^1^	-17.47	1.01	-0.72	-17.24	**< 0.001**	-0.47	-0.58	-0.59
	Antisocial/Psychoticism^1^	-11.89	1.09	-0.45	-10.93	**< 0.001**	-0.13	-0.37	-0.42
Fit	R^2^ = 0.36, F(3,564) = 105.51, *p* < 0.001								
Block 2	(Constant)	21.90	2.26		9.67	**< 0.001**			
	OC^1^	-7.45	1.01	-0.31	-7.37	**< 0.001**	0.12	-0.25	-0.30
	UC^1^	-17.46	1.01	-0.72	-17.22	**< 0.001**	-0.47	-0.58	-0.59
	Antisocial/Psychoticism^1^	-11.38	1.10	-0.43	-10.36	**< 0.001**	-0.13	-0.35	-0.40
	Age	0.22	0.10	0.08	2.31	**0.021**	0.15	0.08	0.10
	Female^2^	1.80	0.83	0.08	2.18	**0.029**	0.01	0.07	0.09
	Gender Diverse^2^	0.91	2.82	0.01	0.32	0.747	0.05	0.01	0.01
Fit	R^2^ = 0.37, F(6,561) = 55.27, *p* < 0.001.								
Difference	Δ R^2^ = 0.01, 0.014								

Note. ¹ Reference category = Resilient group. ² Reference category = being male. *b* = unstandardized regression estimate, *beta* = standardized regression estimate, *SE* = standard error, *r* = correlation coefficient. ***Bold*** denotes a significant relationship

As displayed in Table 5, being in the Maladaptive OC, Maladaptive UC or Antisocial/Psychoticism clusters were found to significantly predict a higher score on the EDE-QS and all three DASS-21 subscales compared to membership in the resilient cluster. All of these relationships remained significant when the influence of age and gender was considered. The Part r indicates that being in the UC cluster added substantially to explaining the variability in the outcomes for all three models, followed by being in the antisocial/psychoticism and OC clusters. Being a woman was found to add significantly to explaining the variability in the outcome compared to being a man for the EDE-QS and the Stress DASS-21 subscale.

## Discussion

The overall aim of this research was to understand the OC and UC multi-dimensional construct for the first time using the AMPD as measured by a brief publicly available tool, the PID-5-SF. Consistent with previous research, OC, UC and resilient/low-psychopathology clusters were distinguishable in the data [6, 10, 11, 14–16]. A fourth cluster characterised by antisocial and narcissistic traits was additionally identified within our sample. According to Lynch’s theory [7], each individual in the population would biologically lean towards OC or UC, with the adaptive representation of each type considered a resilient or flexible presentation. There is a possibility that the four clusters identified in this study represent both adaptive and maladaptive representations of both OC and UC. To our knowledge, our research is the first to focus on using a measure of the AMPD personality traits to distinguish personality types, paving the way for future research to potentially bridge the gap between contemporary perspectives on personality pathology and clinical research on treating personality types. We will discuss each of the clusters separately.

The OC cluster consisted of just over one quarter of the sample and was associated with elevated scores on dimensions that are representative of OCPD and Avoidant PD. Our finding is consistent with the expectations of this study, and theorised OC personality disorders as outlined by Lynch [7]. Additionally, the OC cluster were found to endorse frequent hopelessness and low mood, in addition to mood swings. Although these traits are traditionally associated with borderline personality disorder (which is considered a UC personality disorder according to Lynch [7], as mentioned earlier), they represent the emotional experience of an individual which may be expressed differently by OC and UC types. More specifically, OC is more likely to be associated with internalised expression, and UC with externalised expression [61, 62]. Although the pattern of elevations observed in the OC cluster are consistent with the theoretical perspectives, it is important to note that the UC cluster (explored below) also achieved high scores on most of the same PID-5 subscales. What distinguished the OC cluster from the UC cluster is the pattern of low scores. In particular, low scores on traits associated with narcissism and antisocial PD provide support for theoretical constructs of OC of disliking attention from others and a tendency toward interpersonal avoidance [8, 6, 10, 11, 15]. OC has previously been linked with low impulsivity [8, 3, 6, 63] and this was also evident in our data.

Then maladaptive UC cluster was associated with higher scores than other clusters on both personality and clinical scales, with just under one quarter of the sample falling in this group. The UC group demonstrated a response pattern to self-report measures consistent with previous observations that a UC group has a tendency to consistently endorse traits [62], A high-psychopathology cluster has been previously identified in three cluster solutions among individuals with eating disorders [64] and substance use disorders [65]. Our findings are consistent with previous findings that the UC cluster is generally associated with higher levels of psychopathology and dysfunction compared to the OC and resilient clusters, and our research supports this [8, 3, 6, 7, 10, 11, 13, 15, 63, 66, 67].

Cluster 3 was characterised by low scores on all measured subscales and was named the resilient cluster, comprising of just over one quarter of the total sample. Past research has consistently identified a high functioning/low psychopathology cluster [6, 10, 11, 66–69]. In our study, the resilient cluster was characterised by low scores on all personality and clinical scales, and a smaller proportion of individuals who achieved EDE-QS scores that might be indicative of an eating disorder. Past research has indicated that while being in the resilient cluster appears to be protective against having a clinical disorder [9, 66], and those resilient individuals who do develop a clinical disorder have been found to have a more positive prognosis and response to treatment [6, 14].

The fourth and final cluster we proposed the named the “antisocial/psychoticism” cluster as it was characterised by elevated scores on the attention seeking, callousness, deceitfulness, intimacy avoidance, manipulativeness, restricted affectivity and risk-taking subscales. This was also the smallest cluster, making up approximately one-fifth of the total sample. Interestingly, nearly half of the participants in the antisocial/psychoticism identified as men which may potentially indicate gender differences in the way personality type presents, but further research is needed. The cluster demonstrated some elevation on facets within the psychoticism domain compared to the resilient and overcontrolled cluster. Although there is some resemblance between our Antisocial/Psychoticism cluster and the behaviourally dysregulated cluster identified by Thompson-Brenner et al. [14] or elements of the severe cluster identified by Goldner and their team [11], the presence of a cluster characterised by antisocial and narcissistic traits is unusual in the existing literature. However, as there is little research in this area, further exploration is required.

### Personality types and clinical symptoms

The second aim of our research was to understand the relationships between personality type and clinical symptoms, particularly eating pathology, depression, anxiety and stress. The proposed OC, UC and antisocial/psychoticism clusters scored significantly higher on measures of eating pathology, depression, anxiety and stress than the resilient cluster, consistent with expectations of our study and past research [9, 66]. The finding that the UC group demonstrated the highest ED risk was contradictory to previous research using a combined clinical and non-clinical sample [12]. However, the UC type has been previously linked to higher binge eating presentations, and EDs associated with binge eating behaviour presented at a higher prevalence in the community [70] our finding can therefore be considered unsurprising.

Our results indicated a higher incidence of clinical ED scores in the OC, UC and antisocial/psychoticism groups compared to the resilient group, and presence in the OC, UC and Antisocial/Psychoticism cluster was found to predict eating pathology, depression, anxiety and stress. Although being a woman added significant predictive value to the eating pathology model, and being a woman at the older end of our age range were significant predictors of stress, cluster membership remained significant predictors. Taken together, our results are consistent with the expectations of our study, in addition to past research, that has linked increased clinical pathology to OC and UC groups [9, 66]. Our results also indicated that elevated scores on self-reported clinical measures in the antisocial/psychoticism, group compared to the resilient group, but generally lower than scores for OC and UC groups. The Antisocial/Psychoticism demonstrated higher scores on measures of disordered eating, eating pathology, depression, anxiety, and stress than the resilient group, however lower scores on measures of body image dissatisfaction, depression and stress than the OC and Resilient groups. Taken with the observations that Mean scores on clinical scales were found to fall within mild to moderate ranges, and less than one-third of the participants in this cluster achieved EDE-QS scores indicative of eating pathology, it might be implied that this group would be less likely to be distinguished in clinical settings, or in clinical samples. Our previous research identified a novel link between UC personality type and negative attitudes towards obesity [9], which was reflected in higher negative attitudes towards obesity scores in the UC and Antisocial/Psychoticism groups. The Antisocial/psychoticism cluster was further characterised by higher scores on excessive exercise and muscle building. Our finding is consistent with previous studies have identified a relationship between constricted emotional expression, risk taking and social insensitivity with exercising and muscle building behaviour [34, 71]. Chewing and spitting behaviour has previously been linked to impulsivity [72], and our research lends further support for this. In general, the results of this study provide further support for previous findings that externalising behaviour may be related to a tendency to externalise appearance ideals.

### Limitations

There are some limitations that should be taken into account when interpreting our results. Firstly, the majority of the sample were Caucasian which may limit the generalisability of our results to other ethnic groups, as differences have been found in ED presentations among differing ethnic backgrounds [73]. Likewise, ED presentations have been found to differ between men and women [33, 34, 74]. Our research included more women than men or gender diverse participants, and while we did control for gender in our regression analyses, there may be some limitations of the generalisability of our results to men and gender diverse individuals. Thirdly, the data in the current sample was collected in Australia during the COVID-19 pandemic. Research has indicated increased psychological distress and ED behaviours associated with the pandemic [75]. Therefore, we are uncertain that results regarding measures of psychological distress would differ if the data was collected with a sample of post-pandemic young Australians. Finally, in spite of the benefits of using self-report measures, there are several limitations. In particular, there are concerns that individuals with personality disorder may not be able to accurately describe personality traits, that individuals may be able to distort their presentation and there may be limited insights into the problematic aspects of personality functioning [76]. As a result, it is important to consider our results within these limitations.

### Implications and areas for future research

Our results add to the existing literature regarding personality types. To our knowledge, this is the first study to explore the personality types construct using the pathological personality traits of the AMPD. It can be implied is that the PID-5-SF, a measure of the AMPD that has been found to be efficacious in identifying DSM-5 categorical personality disorders [22–29], may also be a useful measure to identify personality types. It is likely that having one measure that can assess traits, disorders and types will be preferable due to being parsimonious for both clinicians and clients, however more research is needed to confirm appropriateness of use in clinical samples. Second, we found significant differences in disordered eating behaviours and clinical pathology between our clusters, which potentially adds further support to the literature regarding the relationship between personality types and clinical behaviour, strengthening arguments that classifying individuals with EDs based on personality type may demonstrate superior clinical utility compared to diagnostic categories [5, 6].

Although there has previously been extensive research on the relationship between personality types and EDs, our research is unique in multiple ways. First, the focus on personality types in non-clinical samples is currently limited [9, 66]. It is possible that identifying the Antisocial/Psychoticism cluster was a result of considering a broader, more representative sample for our analyses. Second, the use of a measure of the AMPD pathological personality traits provided us with the opportunity to understand the personality types construct using a model of personality pathology that is associated with a contemporary understanding of personality pathology (Busch, Morey, & Hopwood, 2017; Skodol, 2012; Thylstrup et al., 2016. Therefore, further research will be needed to replicate our results in other samples. In addition, it is likely that further research will be necessary to validate the PID-5-SF subscales that identified OC in clinical samples, and to identify if personality types identified using the PID-5-SF subscales can predict treatment outcome, consistent with previous research [6, 14].

## Conclusions

The overall aim of the current research was to explore the emergence of personality types using a measure of the APMD among a sample of adolescent and young adult men and women. To our knowledge, our results were the first to demonstrate that clusters consistent with OC, UC and resilient types could potentially be identified using AMPD pathological personality traits. We also proposed an additional cluster that appeared to be characterised by antisocial and narcissistic traits and mild clinical pathology. Our research helps to provide an additional step towards potentially assessing and understanding OC and UC types in clinical practice. In general, the results of the current study appear to support assertions of the need to understand disordered eating behaviour in the context of personality types and pathology to possibly enhance the formulation of potentially problematic disordered eating behaviour and opened avenues for future research.

## Data Availability

The datasets generated and analysed during the current study are not publicly available as ethics approval was granted under the circumstances of complete confidentiality of participant data.
